# Probing the origin of the giant magnetic anisotropy in trigonal bipyramidal Ni(ii) under high pressure†
†Electronic supplementary information (ESI) available. CCDC 1579468–1579472. For ESI and crystallographic data in CIF or other electronic format see DOI: 10.1039/c7sc04460g


**DOI:** 10.1039/c7sc04460g

**Published:** 2017-12-19

**Authors:** Gavin A. Craig, Arup Sarkar, Christopher H. Woodall, Moya A. Hay, Katie E. R. Marriott, Konstantin V. Kamenev, Stephen A. Moggach, Euan K. Brechin, Simon Parsons, Gopalan Rajaraman, Mark Murrie

**Affiliations:** a WestCHEM , School of Chemistry , University of Glasgow , Glasgow , G12 8QQ , UK . Email: mark.murrie@glasgow.ac.uk; b Department of Chemistry , Indian Institute of Technology Bombay , Powai , Mumbai , Maharashtra 400 076 , India . Email: rajaraman@chem.iitb.ac.in; c Centre for Science at Extreme Conditions , University of Edinburgh , Edinburgh , EH9 3FD , UK . Email: Simon.Parsons@ed.ac.uk; d EaStCHEM , School of Chemistry , University of Edinburgh , Edinburgh , EH9 3FJ , UK

## Abstract

Understanding and controlling magnetic anisotropy at the level of a single metal ion is vital if the miniaturisation of data storage is to continue to evolve into transformative technologies. Magnetic anisotropy is essential for a molecule-based magnetic memory as it pins the magnetic moment of a metal ion along the easy axis. Devices will require deposition of magnetic molecules on surfaces, where changes in molecular structure can significantly alter magnetic properties. Furthermore, if we are to use coordination complexes with high magnetic anisotropy as building blocks for larger systems we need to know how magnetic anisotropy is affected by structural distortions. Here we study a trigonal bipyramidal nickel(ii) complex where a giant magnetic anisotropy of several hundred wavenumbers can be engineered. By using high pressure, we show how the magnetic anisotropy is strongly influenced by small structural distortions. Using a combination of high pressure X-ray diffraction, *ab initio* methods and high pressure magnetic measurements, we find that hydrostatic pressure lowers both the trigonal symmetry and axial anisotropy, while increasing the rhombic anisotropy. The ligand–metal–ligand angles in the equatorial plane are found to play a crucial role in tuning the energy separation between the d_*x*^2^–*y*^2^_ and d_*xy*_ orbitals, which is the determining factor that controls the magnitude of the axial anisotropy. These results demonstrate that the combination of high pressure techniques with *ab initio* studies is a powerful tool that gives a unique insight into the design of systems that show giant magnetic anisotropy.

## Abstract 2

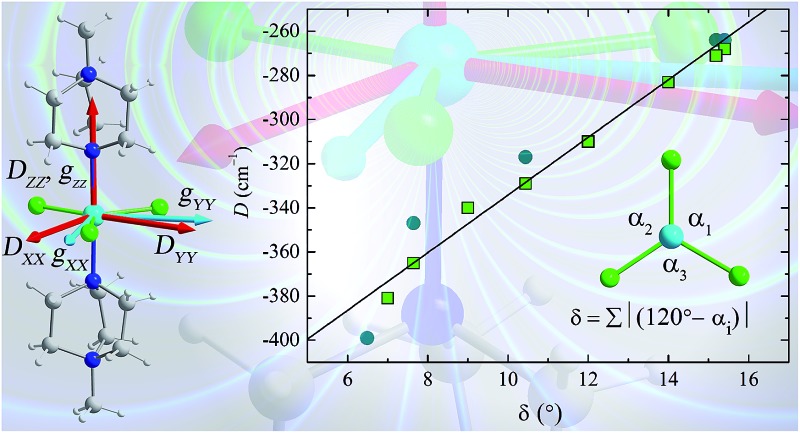
The synergistic combination of high pressure techniques with *ab initio* methods creates a powerful tool to understand giant magnetic anisotropy.

## Introduction

A crucial feature of molecule-based magnets for information storage and spintronic applications is the presence of giant axial magnetic anisotropy.^1^ The barrier to magnetic relaxation, which would cause loss of data, is determined by the size of the spin ground state and the size of the axial anisotropy.^2^ However, ultimately it is the axial magnetic anisotropy that will pin the magnetic moment of the spin ground state (regardless of its size) along one direction or the other of the *z*-axis, allowing the exploitation of these materials in technological applications with consequent increases in the density of data storage. This realisation has been the driving force behind the field of single-ion magnets (SIMs).^3^,^4^ Here, the focus has been on achieving exquisite control of the coordination environment around a single paramagnetic metal ion, to generate a ligand field that leads to first order spin–orbit coupling (SOC). This strategy has been applied to both lanthanide^3^ and transition metal ions.^4^ For lanthanide metal ions, this approach recently led to large easy-axis magnetic anisotropy and observation of magnetic hysteresis at temperatures as high as 60 K for a mononuclear dysprosocenium complex ([Dy(Cp^ttt^)_2_][B(C_6_F_5_)_4_], where Cp^ttt^ is a cyclopentadienyl derivative),^5^ which represents the biggest single improvement in molecular magnet performance since the field began.^6^ Meanwhile, magnetic bistability was recorded at 29 K for the compound [K(crypt-222)][Fe(C(SiMe_3_)_3_)_2_], containing the transition metal ion Fe(i).^7^ Smaller still, work involving the deposition of single atoms of Co or Ho onto ultrathin layers of MgO(100) surfaces has revealed magnetic anisotropies approaching the theoretical limit for the transition metal,^8^ and magnetic bistability for the rare earth.^9^ For all of these examples, the large magnetic anisotropy arises from achieving highly axial coordination environments.

**Fig. 1:**
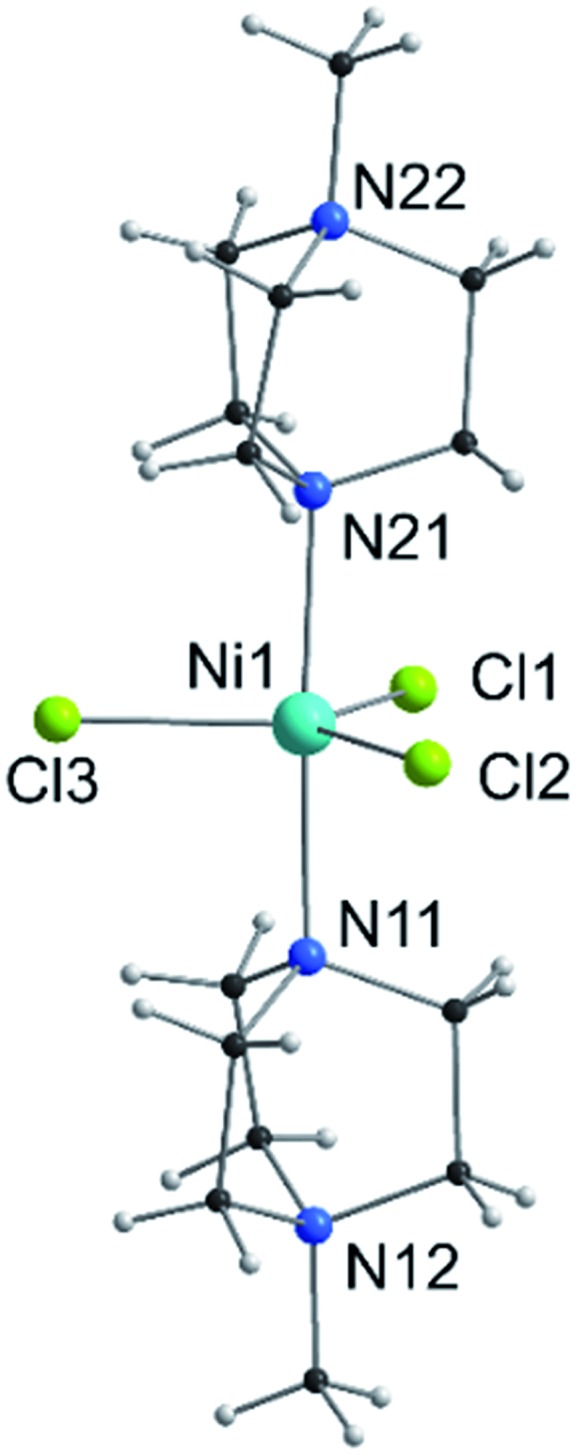
View of the molecular structure of the [Ni(MeDABCO)_2_Cl_3_]^+^ cation in **1**. Only heteroatoms are labeled.

Giant magnetic anisotropy was predicted on the basis of gas phase calculations for a simulated complex of the type [Ni(MeDABCO)_2_X_3_]^+^, where X^–^ is a halide ion and MeDABCO is the cationic ligand 1-methyl-4-aza-1-azoniabicyclo[2.2.2]octanium.^10^ The bulky MeDABCO ligand was expected to minimise structural distortions that would arise from the Jahn–Teller effect. These distortions would lift the degeneracy of the d_*xy*_ and d_*x*^2^–*y*^2^_ orbitals in this d^8^ trigonal bipyramidal complex and quench the first order SOC that would otherwise yield a very large axial magnetic anisotropy.^11^ We experimentally confirmed the presence of giant magnetic anisotropy in the compound [Ni(MeDABCO)_2_Cl_3_](ClO_4_) (**1**) through magnetic measurements, and high- and low-field electron paramagnetic resonance (EPR) studies performed on both oriented single crystals and powder samples of **1** (the molecular structure of the cation is shown in Fig. 1).^12^ Even so, the axial magnetic anisotropy was found to be so large that it was not possible to directly determine its magnitude on the basis of high-field, high-frequency EPR measurements, for which best fits of the data suggested that the axial zero-field splitting (ZFS) parameter |*D*| could not be lower than 400 cm^–1^. Given the dependence of magnetic anisotropy on the coordination environment around the metal ion, we were interested in probing (i) whether we could use hydrostatic pressure as a means of inducing changes to the coordination sphere around Ni(ii) in **1** and (ii) the effect this would have on the anisotropy. The application of hydrostatic pressure is becoming a more convenient tool to unveil unusual properties in coordination complexes.^13^ Previously, we have used pressure to increase the magnetic ordering temperatures in mononuclear Re(iv) complexes,^14^ and to control the orientation of Jahn–Teller axes in polymetallic complexes.^15^

Herein, we use single crystal X-ray diffraction to observe pressure-induced modifications to the symmetry around the Ni(ii) ion in **1**. These high pressure experimental structural data were used for state averaged complete active space self-consistent field (SA-CASSCF) calculations to predict the effect of the structural modifications on the relative energies of the 3d orbitals in **1** and thus extract the anticipated changes in the magnetic anisotropy. How magnetic anisotropy is influenced by small structural distortions is an important question with wide implications, as deposition of magnetic molecules on surfaces has been shown to lead to structural alterations that induce drastic changes in their magnetic properties.^16^ Finally, we use these results to account for the changes we observe in the DC magnetic properties of **1** upon performing high pressure magnetometry. We find that high pressure drives a loss in trigonal symmetry and axiality around the Ni(ii) centre in **1**, with a resulting decrease in the magnitude of the axial ZFS and a concomitant increase in the rhombic ZFS, given by the parameter *E*. These results illustrate the sensitivity of giant magnetic anisotropy to changes in the coordination environment. They also demonstrate the usefulness in applying high pressure techniques to experimentally access structures that cannot be synthesised in the laboratory, allowing their subsequent theoretical study and measurement of their physical properties.

## Results and discussion

### Molecular structure of **1** at ambient pressure

Compound **1** is composed of the cation [Ni(MeDABCO)_2_Cl_3_]^+^ and a charge balancing perchlorate anion. The compound crystallises in the orthorhombic space group *Pca*2_1_ (Table 1), the asymmetric unit contains one of these cation–anion pairs, and the unit cell contains four such pairs overall. None of the four [Ni(MeDABCO)_2_Cl_3_]^+^ cations in the unit cell are mutually aligned (see Fig. S1 in the ESI†). There are no hydrogen bonding interactions, the perchlorate anion displaying short contacts with the methylene groups on the arms of the MeDABCO ligands. The [Ni(MeDABCO)_2_Cl_3_]^+^ cation has two MeDABCO^+^ ligands coordinated to a Ni(ii) ion in the axial positions through the non-methylated nitrogen atoms, and three chloride ligands coordinated in the equatorial positions, such that the geometry around the metal ion is trigonal bipyramidal. The average Ni–Cl bond length is 2.300(1) Å, and the average axial Ni–N bond length is 2.222(3) Å. The coordination geometry is further distorted away from ideal *D*_3h_ symmetry as shown by the bond angles around the Ni(ii) ion. The *trans*-N11–Ni1–N21 angle is bent from 180° to 177.1(1)°, and all of the equatorial *cis*-Cl–Ni–Cl bond angles deviate from 120°: 123.2(1), 119.0(1), and 117.7(1)°, for Cl1–Ni1–Cl2, Cl1–Ni1–Cl3, and Cl2–Ni–Cl3, respectively. These structural distortions arise from the Jahn–Teller effect, splitting the d_*xz*_ and d_*y*z_ orbitals, as well as the d_*x*^2^–*y*^2^_ and d_*xy*_ orbitals (*vide infra*), which are degenerate in strict *D*_3h_ symmetry. This lifts the orbital degeneracy associated with the ground term, reducing the first-order SOC contribution to the axial magnetic anisotropy. To determine whether it was possible to physically tune the coordination environment around the Ni(ii) centre and therefore the relative energies of the d-orbitals, we used high pressure X-ray crystallography.

**Table 1:** Selected crystallographic data for compound **1**. The ambient pressure data were collected for a single crystal mounted on a Kapton loop, while the high pressure data were collected on a single crystal in a diamond anvil cell under hydrostatic pressure. See ESI Table S1 for unit cell data collected at higher pressures

Pressure/GPa	Ambient	0.58	0.90	1.40	1.65
*λ*/Å	0.71073				
*T*/K	293				
Crystal system	Orthorhombic				
Space group	*Pca*2_1_				
*a*/Å	12.5175(1)	12.3181(7)	12.2089(9)	11.9968(11)	11.9924(11)
*b*/Å	13.0820(1)	12.8429(7)	12.7469(8)	12.5527(11)	12.5546(11)
*c*/Å	13.0989(1)	13.0380(4)	12.9686(5)	12.8642(6)	12.8611(6)
*V*/Å^3^	2145.0(4)	2062.61(17)	2018.2(2)	1937.2(3)	1936.4(3)
*Z*	4				
*D*_calc._/g cm^–3^	1.607	1.671	1.708	1.779	1.780
Reflections	17 781	6211	6037	5214	4999
Unique data	4863	1738	1740	1583	1596
*R*_int_	0.027	0.029	0.030	0.037	0.035
*R*	0.029	0.029	0.031	0.046	0.044
*R*_w_	0.062	0.071	0.052	0.069	0.070
*S*	0.99	1.04	1.00	1.00	0.99
Flack param.	0.012(14)	0.008(15)	0.014(15)	–0.01(2)	0.02(2)
*ρ*_max_, *ρ*_min_/eÅ^–3^	0.39, –0.36	0.22, –0.19	0.31, –0.31	0.68, –0.92	0.58, –0.80

### Molecular structure of **1** at high pressure

**Fig. 2:**
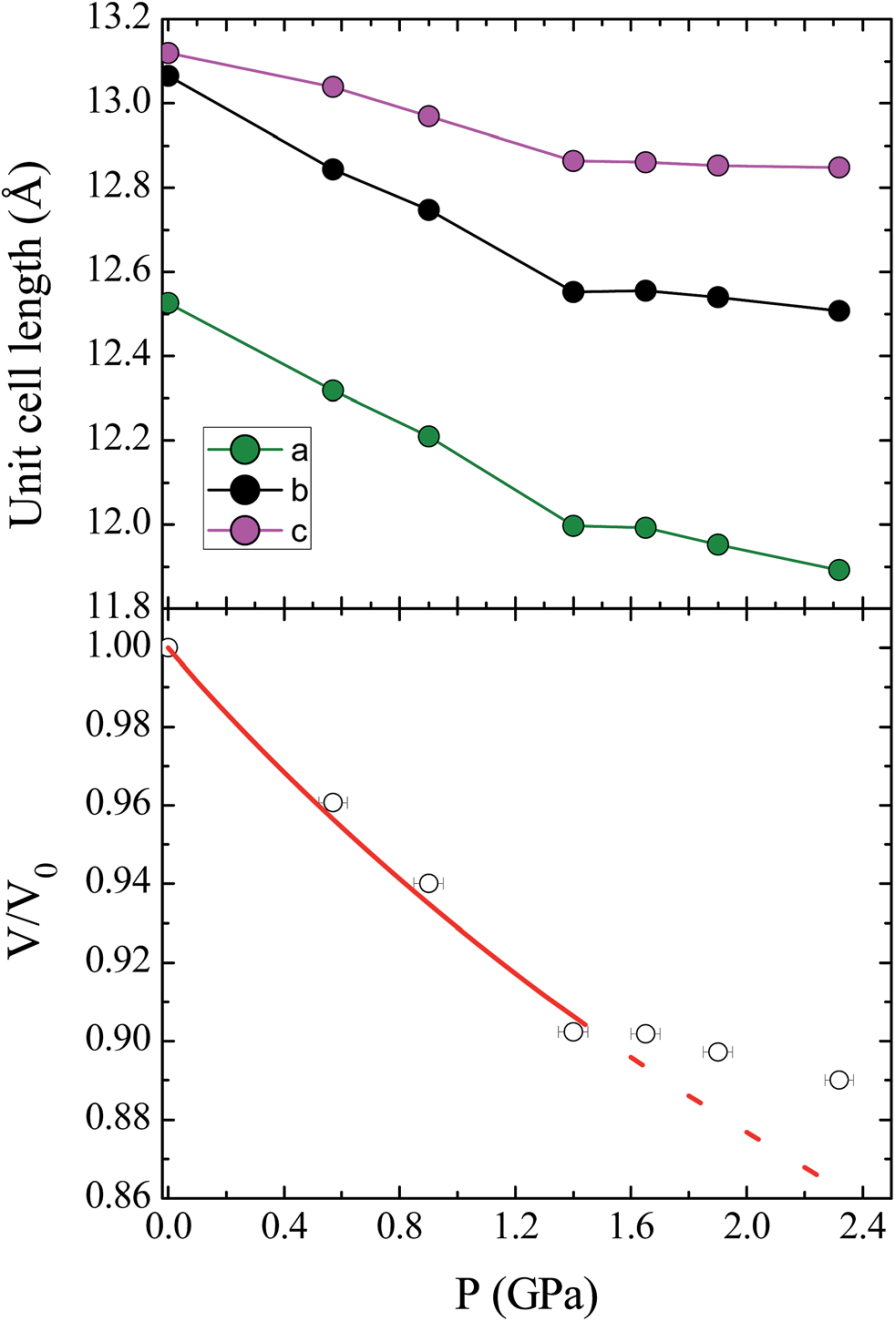
(Top) Contraction of the unit cell lengths in compound **1** with pressure. (Bottom) Pressure dependence of the relative unit cell volume, *V*/*V*_0_, as a function of pressure. The empty circles represent experimental data, and the solid line represents the fit to a second-order Birch–Murnaghan equation of state. The dashed line represents the continuation of the fit, illustrating the change in compressibility of **1** at high pressures (see ESI† for details).

Unit cell parameters for compound **1** were determined over the pressure range 0.58–3.51 GPa, while the crystal structure of **1** was determined at four high pressure points over the range 0.58–1.65 GPa, within the hydrostatic limit of the pressure transmitting medium Fluorinert FC-77.^17^ Under these conditions the compound remains in the *Pca*2_1_ space group, with no major changes to the relative orientations of the molecules in the lattice. The unit cell shows a monotonic decrease in the cell volume with pressure until 1.40 GPa, where there is a change in the compressibility of the lattice (Fig. 2; see ESI† for discussion of the change in compressibility). The pressure dependence of the unit cell volume up to 1.40 GPa could be fitted to a second-order Birch–Murnaghan equation of state, using the program EoSFit7.^18^ This process yielded a value for the bulk modulus *K*_0_ = 11.7(6) GPa, which is a typical value for this type of molecular solid.^19^

**Fig. 3:**
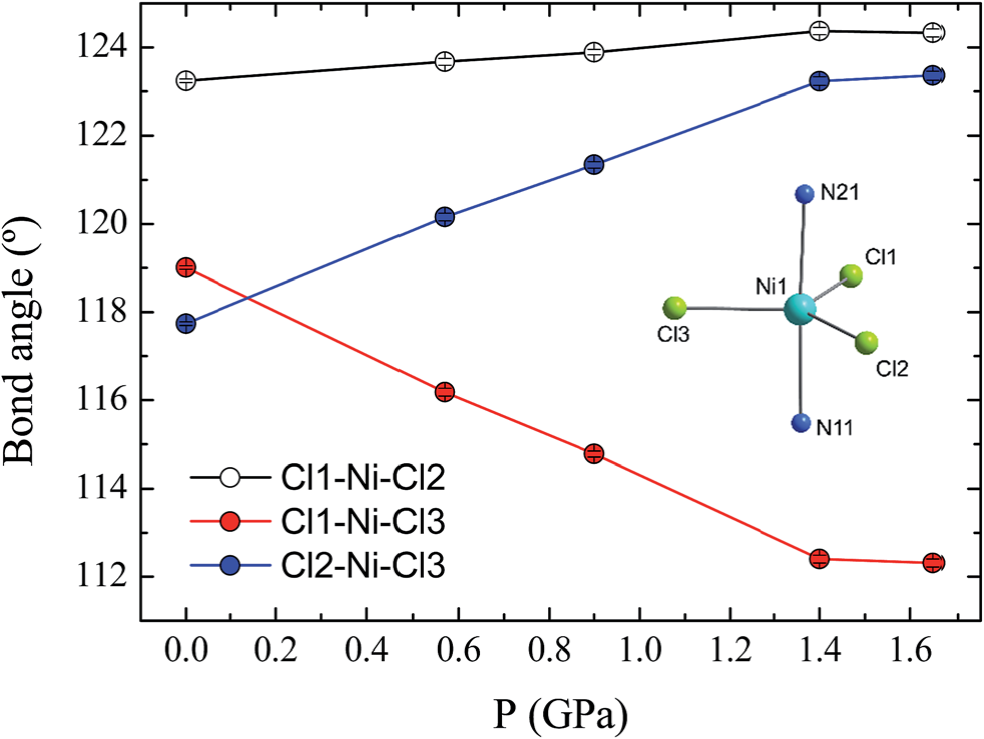
The variation in the equatorial Cl–Ni–Cl bond angles in compound **1** as a function of pressure. The error bars are shown, but are smaller than the symbols. The inset shows the coordination sphere of the Ni(ii) ion in **1**.

The effect of applying pressure on the bond lengths around the Ni(ii) ion in **1** is negligible. The Ni–Cl bonds in the equatorial plane are found to be insensitive to pressure, while there is a very slight compression in the axial Ni–N bonds, which decrease in length from 2.222(3) to 2.194(6) Å at 1.65 GPa (see ESI, Fig. S3†). In stark contrast, there is a significant deformation of the equatorial bond angles around the Ni(ii) ion (Fig. 3). As pressure is applied, the angles formed by Cl1–Ni1–Cl2 and Cl2–Ni1–Cl3 increase, reaching values of 124.3(1) and 123.4(1)°, respectively, at 1.65 GPa, while at the same time the Cl1–Ni1–Cl3 angle decreases to 112.3(1)°, along with a slight decrease in the *trans*-N–Ni–N angle, from 177.1(1) to 176.2(2)° (Fig. S4†). The result of the pressure-induced angular deformations is a lowering of the symmetry around the Ni(ii) ion. Continuous shape measures, which compare the symmetry of the environment around an atom to ideal reference polyhedra,^20^ can be used to quantify the observed symmetry lowering: for compound **1**, *S*(*D*_3h_) = 0.09 at ambient pressure, while at 1.65 GPa, *S*(*D*_3h_) = 0.23, where larger values indicate lower symmetry and *S*(*D*_3h_) = 0 would signify ideal *D*_3h_ symmetry. In an earlier theoretical study regarding the magnetic anisotropy of this type of trigonal bipyramidal Ni(ii) complex at ambient pressure, the importance of controlling these angular distortions to avoid the quenching of first order spin–orbit coupling was highlighted for a series of simulated complexes.^10^ We used these new high pressure structural data to perform *ab initio* calculations on **1** to extract the ZFS parameters *D* and *E* associated with the distinct symmetry observed at each pressure point.

### Theoretical study of the ambient pressure structure of **1**

The zero-field splitting parameters in transition metal complexes are determined by the following spin-Hamiltonian^21^1
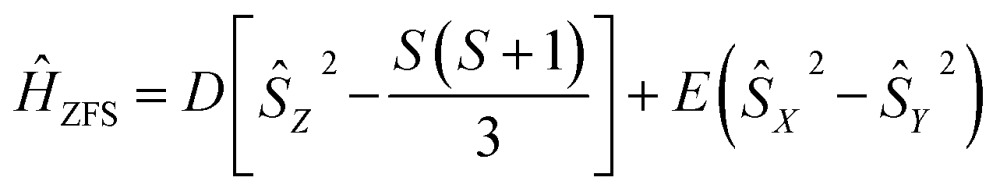
where *D* is the axial ZFS parameter, *E* is the rhombic ZFS parameter and *S*, *S*_*X*_, *S*_*Y*_ and *S*_*Z*_ are the total spin and its *x*, *y*, and *z* components respectively. The overall *D* is a tensor quantity and if this is made diagonal and traceless then its components can be written as^21^2
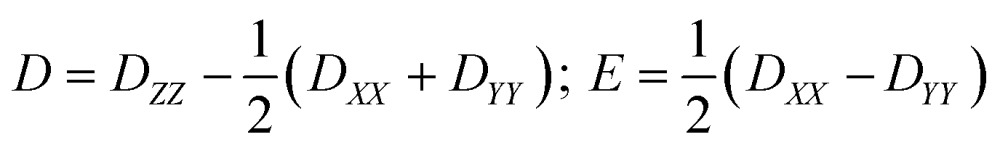



The components of *D* (say, *D*_*ij*_ in general) are themselves negative from the equation derived from second-order perturbation theory,^22^3
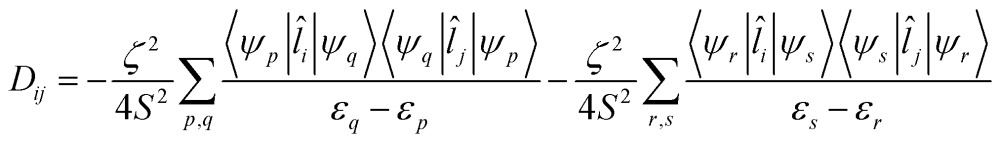



Therefore, *D* is negative when the *D*_*ZZ*_ term becomes greater than the average of the *D*_*XX*_ and *D*_*YY*_ terms. The *D*_*ZZ*_ term in turn becomes dominant when some *M*_L_ level electronic transitions take place as shown in eqn (3). In eqn (3), *ζ* is the effective spin–orbit coupling constant of the molecule, whereas *ε*_p_, *ε*_r_ and *ε*_q_, *ε*_s_ are the energies of the ground and corresponding excited states, respectively. The first term of eqn (3) corresponds to spin allowed β → β electronic transitions from the *ψ*_p_ MO to the *ψ*_q_ MO and the second term corresponds to spin-allowed α → α electronic transitions from the *ψ*_r_ MO to the *ψ*_s_ MO. Furthermore, *l*_*i*_ and *l*_*j*_ are the *x*, *y* or *z* components of the total orbital angular momentum operator *L*, which connects the corresponding ground state wavefunction with the excited state.

**Fig. 4:**
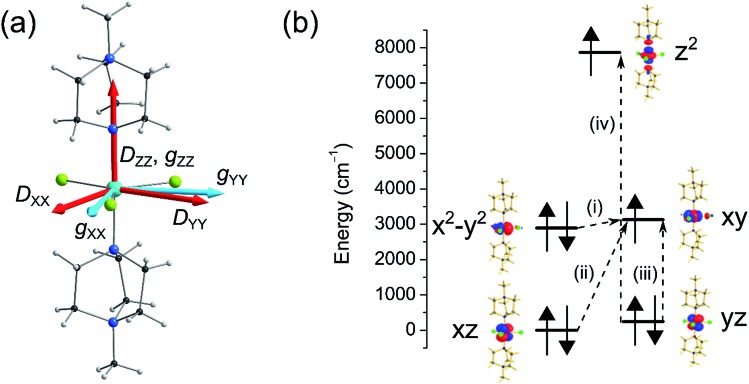
(a) Crystal structure of the cationic complex in **1** along with the orientations of the *D* axes (in red) and *g* values (in light blue; the *g*_*ZZ*_ axis coincides with the *D*_*ZZ*_ axis). (b) NEVPT2-LFT computed d-orbital energies of the Ni(ii) ion in **1** at ambient pressure along with the most significant excitations that contribute to the total *D* value: (i) –488 cm^–1^; (ii) +22 cm^–1^; (iii) +19 cm^–1^; (iv) +8 cm^–1^.

Here we have employed the CASSCF/NEVPT2 method along with the effective Hamiltonian approach to extract the ZFS parameters. This approach has been found to yield good numerical estimates for several examples studied by us^23^ and others.^21^ We begin our discussion with calculations based on the crystal structure of complex **1** collected at ambient pressure.^12^ CASSCF calculations yield a *D* value of –409 cm^–1^ with *E*/*D* estimated to be 0.0004, while the inclusion of a dynamic correlation yields very similar parameters (*D* = –399 cm^–1^ and *E*/*D* = 0.0003, Tables S2 and S3†). The very large *D* parameter obtained from the calculation is consistent with that estimated previously from broadband high-field EPR studies, where the application of a large magnetic field transverse to the easy-axis enabled an indirect estimation of *D*, with a lower bound set at |*D*| ∼ 400 cm^–1^. The computed anisotropy axes (*D* tensor directions) are shown in Fig. 4. The *D*_*ZZ*_ axis is found to lie along the pseudo-*C*_3_ axis (in the N–Ni–N direction) and the computed *g*_*zz*_ is found to coincide with this axis. From symmetry considerations, a Ni(ii) complex with a d^8^ electronic configuration possessing perfect *D*_3h_ symmetry should have degenerate d_*xy*_ and d_*x*^2^–*y*^2^_ orbitals and hence, a large first-order spin–orbit contribution to the magnetic anisotropy. However, despite the presence of the bulky ligands in the axial positions of the Ni(ii) ion in compound **1**, the symmetry is lowered slightly and a Jahn–Teller distortion breaks this orbital degeneracy. Hence, the use of ZFS to describe the magnetic anisotropy is appropriate, although as we noted previously this description does push the limits of the spin-only model.

In complex **1**, the very large *D* value stems from the closely lying d_*x*^2^–*y*^2^_ and d_*xy*_ orbitals (their separation is estimated to be 239 cm^–1^) which contribute –488 cm^–1^ to the total *D* parameter (see Fig. 4 and Table S4†). We find that there are very small positive contributions from the other excited states which diminish the negative *D* value. Among these transitions, excitations from the d_*xz*_ and d_*yz*_ to the d_*xy*_ orbital are the most important contributions, as shown in Fig. 4. The energy of the first six spin–orbit states with their contribution from the ground state and first excited state are provided in Table S5.† Since *D* is negative, *M*_S_ = ±1 is the ground state followed by the *M*_S_ = 0 state. The first two spin–orbit states consist mostly of the ground triplet state (64%). Most importantly, the tunnel splitting between the *M*_S_ = ±1 states is estimated to be 0.21 cm^–1^, suggesting very fast ground state relaxation. This is consistent with our previous study, where no out-of-phase ac susceptibility signals for **1** were observed in the absence of an applied dc field.^12^ Strong tunnelling for the *M*_S_ = ±1 state is essentially due to the rhombic *E* term as noted in Table 2 (*E* = 0.10 cm^–1^). The main contributions to *E* stem from electronic transitions between different *M*_L_ levels but due to the presence of relatively high symmetry, these cancel each other out leading to a moderately small contribution to *E*. In addition to the *E* term, both the Cl and the N atoms cause hyperfine interactions, which offer an additional pathway for resonant tunnelling and lower the effective barrier to reorientation of the magnetisation, even in the presence of an applied dc field.^12^

**Table 2:** NEVPT2 calculated *D* and *E* values computed from the high pressure single-crystal X-ray data along with the most prominent contribution to *D*, the tunnel splitting of the *M*_S_ = ±1 levels and the *δ* value computed for the structure

Pressure	*D* (cm^–1^)	*E* (cm^–1^)	Contribution from 1^st^ excited state (NEVPT2) (cm^–1^)	Tunnel splitting (cm^–1^)	Sum of Cl–Ni–Cl angle deviation, *δ* (°)
Ambient	–399	0.104	–488	0.21	6.49
0.58 GPa	–347	0.208	–435	0.42	7.64
0.90 GPa	–317	0.419	–403	0.84	10.44
1.40 GPa	–264	0.861	–346	1.72	15.19
1.65 GPa	–264	0.871	–346	1.75	15.4

### Theoretical study of the effect of pressure on the magnetic anisotropy of **1**

To understand how the magnetic anisotropy changes upon application of pressure, we have carried out *ab initio* calculations using the high pressure single crystal diffraction data. The computed ZFS parameters are summarised in Table 2. As discussed earlier, upon increasing pressure the most important structural change that we observe is in the ∠Cl–Ni–Cl angles (*α*), which give a good measure of the extent of the Jahn–Teller distortion.^10^ Consequently changes in *α* are expected to lead to large changes in the *D* values. Our calculations predict a decrease of the *D* value of one third, from –399 cm^–1^ at ambient pressure to –264 cm^–1^ at 1.4 GPa. The variation in *D* of ∼130 cm^–1^ as the pressure is increased to 1.4 GPa highlights the degree of sensitivity of the magnetic anisotropy to structural changes. The application of high pressure overcomes the steric constraints of the bulky ligands, which force **1** to be very close to ideal trigonal bipyramidal geometry, serving to increase the magnitude of the Jahn–Teller distortions.

**Fig. 5:**
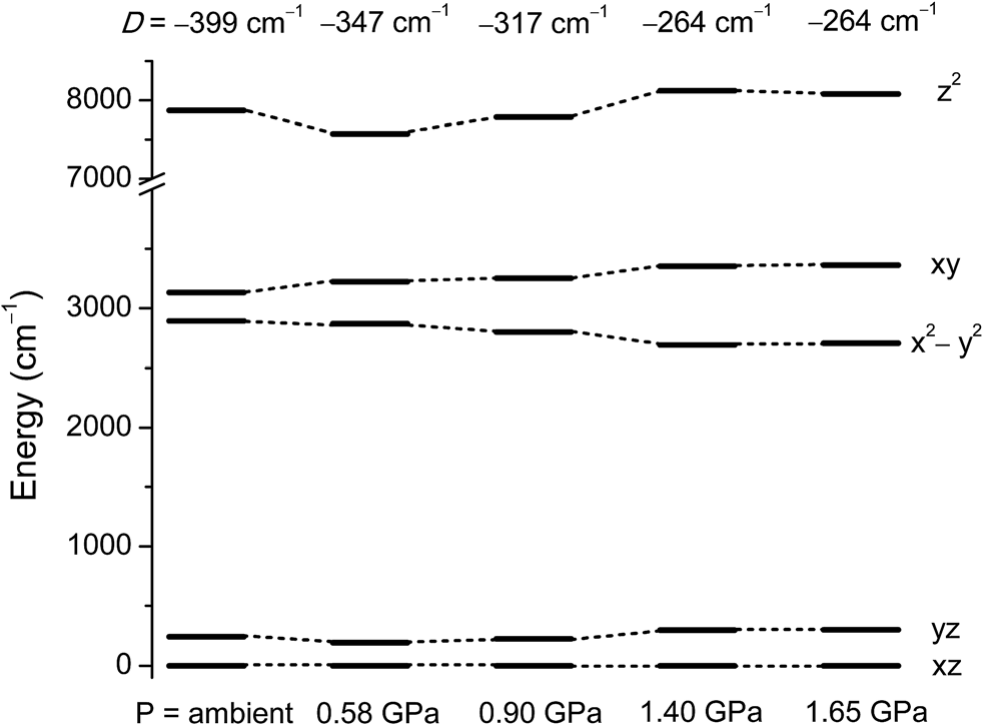
NEVPT2 computed ligand field d-orbital splitting for the 3d orbitals in **1** at the pressure points corresponding to the single crystal X-ray structures.

The decrease in *D* as the pressure increases is essentially due to the deviation in the *α* angles around the equatorial plane, with larger deviations away from 120° leading to a larger separation between the d_*xy*_ and d_*x*^2^–*y*^2^_ orbitals (*vide supra*). As the gap between these two orbitals increases, the associated major contribution to *D* drops significantly, leading to much lower *D* values (Fig. 5 and Table S2 in the ESI†). As the structural changes induced by pressure lead to negligible variations in the contributions to *D* from other excited states, the major change in *D* thus arises from the shift in the relative energies of the d_*xy*_ and d_*x*^2^–*y*^2^_ orbitals. The decrease in the magnitude of *D* with pressure is accompanied by an increase in the rhombic anisotropy *E*, from 0.10 cm^–1^ at ambient pressure to 0.86 cm^–1^ at 1.4 GPa (Table 2). The most significant contribution to the increasing *E* parameter arises from the increasing separation of the d_*xz*_ and d_*yz*_ orbitals with pressure (Fig. 5 and Tables S6–S14 in the ESI†). As *E* increases, the tunnel splitting between the *M*_S_ = ±1 states increases from 0.42 cm^–1^ to 1.72 cm^–1^ at 1.4 GPa. This suggests that as the applied pressure increases, not only does the axial anisotropy decrease, but the increased tunnel splitting will also lead to faster quantum tunnelling of the magnetisation.

**Fig. 6:**
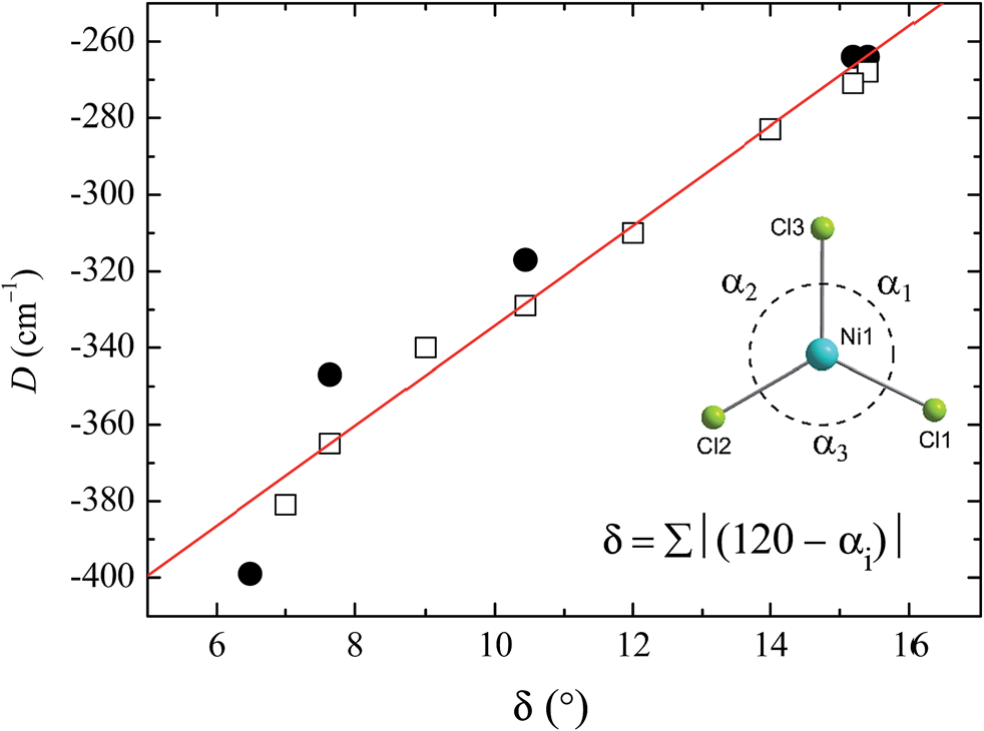
Magneto-structural correlation developed for the *δ* parameter against computed *D* values. The black circles are the calculated *D* values obtained for the X-ray structures collected at high pressure, and the red line is a linear fit. The white squares represent the NEVPT2-computed *D* values, obtained by altering the *δ* value of the X-ray structure of **1** obtained at ambient pressure. (Inset) Definition of *δ*, and a view of the equatorial plane in **1**, with the axial ligands omitted for clarity.

To see clearly how the *α* angles influence the calculated magnitude of *D*, we define the parameter *δ* as the sum of the deviations of each angle *α* from the ideal value of 120° associated with trigonal symmetry (Fig. 6). There is an approximately linear relationship between the size of this structural deviation *δ* and the axial zero-field splitting parameter. To further illustrate the importance of the parameter *δ* in determining the magnetic anisotropy in **1**, and to exclude the possibility that the small changes in other structural features (such as the Ni–N and Ni–Cl bond lengths) have any significant impact on *D*, we calculated *D* for a series of simulated structures of **1** where *δ* was varied while all of the other structural parameters were kept constant. We computed eight points with various *δ* values and their associated *D* values (shown as white squares in Fig. 6). The computed values of *D* for the simulated complexes are close to those calculated for the HP structural data (shown as black circles in Fig. 6), and lie very close to the linear relationship observed between *D* and *δ*, suggesting that the observed variation in *D* is essentially due to *δ* and independent of other structural features.

Overall, the structural data collected point to a lowering of symmetry around the Ni(ii) ion in **1** as high pressures induce a change in the Cl–Ni–Cl equatorial bond angles. The *ab initio* calculations indicate that these structural changes lead to a loss of the axial nature of the ligand field in **1**, with a resulting decrease in the axial ZFS parameter *D*. To determine whether the anticipated changes to the magnetic properties of **1** were accurately described by theory, we performed high pressure magnetic measurements on the compound.

### High pressure magnetic study of **1**

**Fig. 7:**
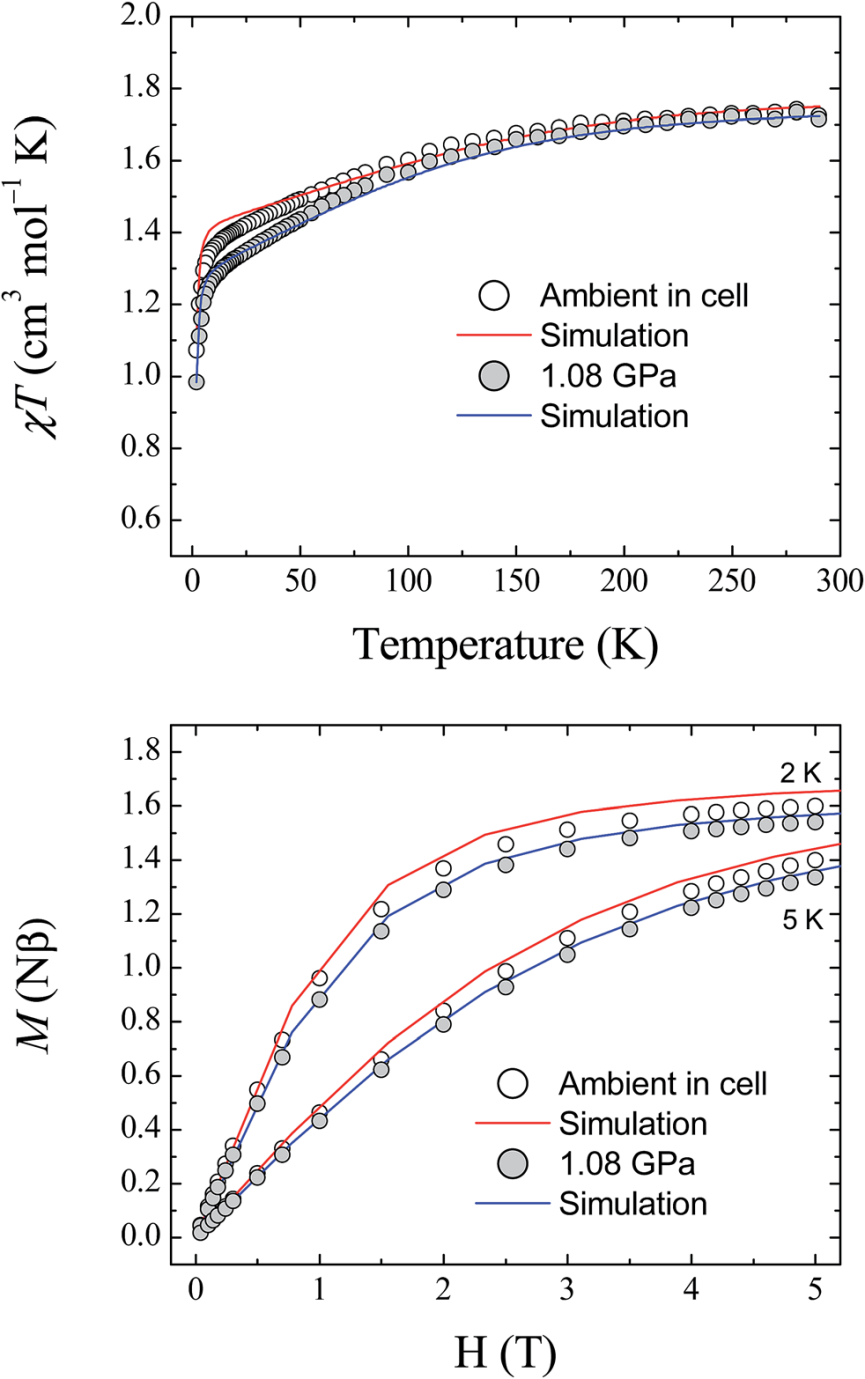
(Top) The temperature dependence of the molar magnetic susceptibility, *χ*_M_*T*, for **1**, measured at ambient pressure and 1.08 GPa. (Bottom) Field dependence of the magnetisation for **1** measured at 2 and 5 K, at ambient pressure and 1.08 GPa. The solid lines represent simulations of the data (red – ambient; blue – 1.08 GPa) using the parameters given in Table 3.

The magnetic properties of **1** were studied using randomly oriented polycrystalline samples in a piston-cylinder pressure cell (see Experimental details). Four pressure points were investigated, ranging from ambient pressure (measured in the cell) to 1.08 GPa. Fig. 7 (top) shows the temperature dependence of the molar magnetic susceptibility product *χ*_M_*T* for **1**, measured over the temperature range 290–2 K, at ambient pressure and at 1.08 GPa, under an applied dc field of 1 T (data collected at 0.52 and 0.79 GPa are provided in the ESI†). At 290 K, the measured values of *χ*_M_*T* are 1.73, 1.68, 1.68, and 1.72 cm^3^ mol^–1^ K for ambient pressure, 0.52, 0.79, and 1.08 GPa, respectively. These values are all close to the value found previously for the polycrystalline sample measured in a gelatin capsule (1.75 cm^3^ mol^–1^ K),^12^ and indicate that a significant orbital contribution to the magnetic susceptibility is present even on increasing the pressure (*χ*_M_*T* = 1.0 cm^3^ mol^–1^ K for an *S* = 1 system where *g* = 2.0). Upon lowering the temperature, *χ*_M_*T* decreases for all four pressure points, with the decrease becoming more pronounced below 100 K before dropping sharply below 10 K. The values measured for *χ*_M_*T* at 2 K are 1.07, 1.04, 1.01, and 0.98 cm^3^ mol^–1^ K for ambient pressure, 0.52, 0.79, and 1.08 GPa, respectively. Additionally, the field dependence of the magnetisation of **1** was measured at 2, 3, 4, and 5 K over the range 0–5 T for each pressure point. Fig. 7 (bottom) shows the data collected at 2 and 5 K for ambient pressure and 1.08 GPa (for clarity the data at 3 and 4 K, and the full data for the remaining pressure points, are given in the ESI†).

Fitting the magnetic data for compound **1** was found previously to be non-trivial because of the large magnetic anisotropy it displays, which requires a highly anisotropic *g*-factor and consequently many parameters.^12^ This leads to a large number of local minima in the fitting process, and thus, it is difficult to reach a unique solution for each pressure point. Therefore, the previously described high field EPR study together with the results of the *ab initio* calculations described here were used as guides to simulate the magnetic data at ambient pressure. As the level of theory employed for the calculations is more suitable for the determination of zero-field splitting parameters than for accurate calculation of *g*-factors, the results yielded by the *ab initio* calculations for *D* and *E* were used directly in the simulation of the magnetic data. The *g*-factors were taken from the previous study,^12^ in which HF-EPR was used to experimentally determine *g*_*z*_ (Table 3). The *χ*_M_*T* data at 1 T and the magnetisation data collected at 2, 3, 4 and 5 K were then simulated using the program PHI (v3.0.6),^24^ following the Hamiltonian:*Ĥ* = *Dŝ*_*z*_^2^ + *E*(*ŝ*_*x*_^2^ – *ŝ*_*y*_^2^) + *μ*_B_*B*·*g*·*ŝ*

**Table 3:** Parameters used for the simulations of the magnetic data shown in Fig. 7 and S5–S10

	*g*_*z*_	*g*_*x*_	*g*_*y*_	*D*/cm^–1^	*E*/cm^–1^
Ambient pressure	3.36	2.05	2.05	–399	0.10
0.52 GPa	3.28	2.12	2.13	–349	0.22
0.79 GPa	3.24	2.16	2.18	–323	0.33
1.08 GPa	3.20	2.20	2.22	–295	0.52

To determine the values of the various parameters for the simulations at high pressure, the pressure dependence of the *ab initio*-calculated parameters was fitted, and used to derive the values for each pressure point used in the magnetic study (the graphs and fits for this process are included in the ESI†). Although the estimations of *D* and *E* are known to be accurate within the reference space chosen for the calculations, reliable estimation of the *g*-tensors requires ligand orbitals to be incorporated in the reference space to fully capture the effect of covalency.^25^ For this reason, the *g*-factors obtained from the *ab initio* calculations were normalised to the *g*-factors found at ambient pressure. The results of this approach are plotted in Fig. 7 at 1.08 GPa (for clarity, the plots for the pressure points at 0.52 and 0.79 GPa are given in the ESI†). Given the giant magnetic anisotropy presented by **1**, and the limitations of the level of theory used in the calculations with respect to the *g* parameters, the results of the *ab initio* study are shown to reasonably describe the effect of applying pressure on the magnetic properties of **1** and show very good agreement with the experimental data.

## Conclusions

In this study, we have shown that hydrostatic pressure can be used to control angular distortions around a trigonal bipyramidal Ni(ii) centre that shows a giant axial magnetic anisotropy. High pressure was found to cause a reduction in the symmetry around the transition metal ion, with the resulting changes driving a drastic decrease in the size of the magnetic anisotropy. By using high pressure crystallography, we have been able to obtain structural data for geometric forms of **1** that do not form under standard conditions. These crystallographic data could then be used to predict the effect of structural distortions on the anticipated magnetic anisotropy for compound **1**, using *ab initio* calculations. The calculations have shown that pressure can be used as an effective tool to control the magnetic anisotropy in compound **1**. We could then complement this structural and theoretical study with high pressure magnetometry, which supported the trends observed in the *ab initio* computed anisotropy parameters. The Cl–Ni–Cl angles (*α*) in the equatorial plane of the Ni(ii) ion were found to play a critical role in tuning the gap between the d_*x*^2^–*y*^2^_ and d_*xy*_ orbitals, which is the determining factor in the size of the axial anisotropy. Calculations performed on model systems revealed that *D* was largely insensitive to all other structural distortions. The results here reveal a new effect of using hydrostatic pressure to modulate the magnetic properties of paramagnetic transition metal complexes, and suggest several open questions, such as whether the reduction of magnetic anisotropy can be avoided by using bulkier anions in the equatorial plane of the Ni(ii) ion, to decrease the compressibility of the *α* angle. One possibility could be the introduction of a compressible moiety into the lattice, capable of absorbing the applied pressure, which was shown to aid the retention of slow magnetic relaxation in a Mn(iii) complex containing Na^+^ ions in the crystal structure.^26^ Another is how a higher symmetry analogue of complex **1** (both in terms of molecular structure and crystal packing) might respond to pressure. Work is under way to investigate these ideas.

## Experimental

Powder and single crystal samples of compound **1** were prepared as described previously.^12^

### Single crystal X-ray diffraction

Single crystal diffraction data for a crystal of **1** mounted on a Kapton loop were collected at room temperature using a Bruker D8 Venture diffractometer. For high pressure studies, a crystal of **1** (0.15 × 0.10 × 0.05 mm^3^) was loaded into a Merrill–Bassett diamond anvil cell (DAC) equipped with 600 mm culet-cut diamonds and conically-ground WC backing plates.^27^ The hydrostatic medium was Fluorinert FC-77, and the pressure was calibrated using a ruby chip. High pressure single-crystal X-ray diffraction data were collected at room temperature on a Bruker SMART APEX II diffractometer with graphite-monochromated Mo Kα radiation (*λ* = 0.71073 Å). The data were integrated using the program SAINT^28^ while employing dynamic masks to account for regions shaded by the pressure cell, and absorption corrections were carried out with SADABS.^29^ The ambient pressure structure was solved using SUPERFLIP.^30^ This solution was used as the basis for the solution of the high pressure data, and all refinements were against *F*^2^ using CRYSTALS.^31^ All non-H atoms were refined anisotropically. For the MeDABCO ligands at high pressure, the bond distances were restrained and the anisotropic displacement parameters of the ligands were subject to similarity restraints. All metal–ligand distances, angles, and torsion angles were refined freely. H atoms were fixed in geometrically calculated positions.

### Computational details

All the first principles calculations were performed using the ORCA 4.0.0 package.^32^ We employed the def2-TZVP basis set for Ni and Cl, the def2-TZVP(-f) basis set for N and def2-SVP for the rest of the atoms.^33^ In order to speed up the integral calculations we have used the RI (resolution of identity) approximation along with the given auxiliary basis sets: SARC/J for Ni, Cl and N and def2/J for the rest of the atoms. We employed these orbitals to start the state averaged complete active space self-consistent field (SA-CASSCF) calculations. The active space in this calculation is comprised of eight d-electrons of Ni in five d-orbitals *i.e.* CAS(8,5). Using this active space, we have computed 10 triplet and 15 singlet roots in the CI (configuration interaction) procedure. To incorporate the dynamic correlation, we employed N-electron valence perturbation theory (NEVPT2) on top of the CASSCF wave function. The def2-TZVP/C for Ni, Cl and N and def2-SVP/C auxiliary basis set for other atoms have been used with the Trafostep RIMO approximation. To account for the scalar relativistic effects, the zeroth-order regular approximation (ZORA) method was used both in the Hamiltonian as well as in the basis functions during all calculations. The zero-field splitting parameters (*D* and *E*) were calculated both from second order perturbation theory and an effective Hamiltonian approach (EHA).^34^ The spin–orbit coupling effects were incorporated by using quasi-degenerate perturbation theory (QDPT) approach.^21^,23a

### High pressure magnetometry

Magnetic measurements under hydrostatic pressure were carried out using a Quantum Design MPMS-XL5 SQUID Magnetometer equipped with a 5 T magnet in the School of Chemistry, University of Glasgow. A polycrystalline sample of **1** was loaded into a CuBe piston-cylinder-type high-pressure capsule cell. Daphne 7373 oil was used as a pressure-transmitting medium, and the pressure determined using the superconducting transition of an indium chip present in the cell.^35^ The data were corrected using a background measurement performed using a complete assembled cell that contained no sample of **1**.

## Conflicts of interest

There are no conflicts of interest to declare.

## Supplementary Material

Supplementary information

Crystal structure data
