# Supplementary material for: Probing the origin of the giant magnetic anisotropy in trigonal bipyramidal Ni(ii) under high pressure
Source: Chem Sci. 2017 Dec 19;9(6):1551–9. doi: 10.1039/c7sc04460g (PMC5890327; doi:10.1039/c7sc04460g)

Electronic Supplementary Material (ESI) for Chemical Science.
This journal is © The Royal Society of Chemistry 2017

Supporting Information for

Probing the origin of the giant magnetic anisotropy in trigonal bipyramidal Ni(II) under high pressure

Gavin A. Craig,[a] Arup Sarkar,[b] Christopher H. Woodall,[c,d] Moya A. Hay,[a] Katie. E. R. Marriott,[a] Konstantin V. Kamenev,[c,d] Stephen A. Moggach,[c,d] Euan K. Brechin,[c,d] Simon Parsons,[c,d] Gopalan Rajaraman[b] and Mark Murrie.[a]

[a]WestCHEM, School of Chemistry, University of Glasgow, Glasgow, G12 8QQ (UK)

[b]Department of Chemistry, Indian Institute of Technology Bombay, Powai, Mumbai, Maharashtra,400 076 (India)

[c]Centre for Science at Extreme Conditions, University of Edinburgh, Edinburgh, EH9 3FD (UK)

[d]EaStCHEM, School of Chemistry, University of Edinburgh, Edinburgh, EH9 3JJ (UK)

CONTENTS

Fig. S1. View of the packing in the unit cell of **1**. 4

Fig. S2. Pressure dependence of the unit cell parameters. 4,5

Table S1. Crystallographic data for compound **1**. 6

Fig. S3. Bond lengths in the first coordination sphere of the Ni(II) ion as a function of pressure. 7

Fig. S4. Variation in the *trans*- N−Ni−N bond angle as a function of pressure in compound **1**. 7

Table S2. Calculated pressure dependence of the parameters *D*, *E*, g_x, g_y, and g_z. 8

Table S3. Comparison of the parameters calculated for the magnetic anisotropy in **1** at ambient pressure using the two different levels of theory. 8

Table S4. Details of the major contributing excitations that lead to the *D* and *E* values calculated for compound **1** at ambient pressure. 8

Table S5. Energy of the lowest spin-orbit states for the ambient pressure structure of **1**. 9

Table S6. Computed NEVPT2 transition energies and state-wise contribution to the axial and rhombic ZFS parameters at ambient pressure. 9

Table S7. Details of the major contributing excitations that lead to the *D* and *E* values calculated for compound **1** at 0.58 GPa. 10

Table S8. Computed NEVPT2 transition energies and state-wise contribution to the axial and rhombic ZFS parameters at 0.58GPa pressure. 10

Table S9. Details of the major contributing excitations that lead to the *D* and *E* values calculated for compound **1** at 0.90 GPa. 11

Table S10. Computed NEVPT2 transition energies and state-wise contribution to the axial and rhombic ZFS parameters at 0.90GPa pressure. 11

Table S11. Details of the major contributing excitations that lead to the *D* and *E* values calculated for compound **1** at 1.40 GPa. 12

Table S12. Computed NEVPT2 transition energies and state-wise contribution to the axial and rhombic ZFS parameters at 1.40 GPa pressure. 12

Table S13. Details of the major contributing excitations that lead to the *D* and *E* values calculated for compound **1** at 1.65 GPa. 13

Table S14. Computed NEVPT2 transition energies and state-wise contribution to the axial and rhombic ZFS parameters at 1.65 GPa pressure. 13
Fig. S5. The temperature dependence of the molar magnetic susceptibility, $\chi_M T$, for **1**, measured at 0.52 GPa. 14
Fig. S6. The temperature dependence of the molar magnetic susceptibility, $\chi_M T$, for **1**, measured at 0.79 GPa. 14
Fig. S7. Field dependence of the magnetisation for **1** measured at 2, 3, 4 and 5 K, at ambient pressure. 15
Fig. S8. Field dependence of the magnetisation for **1** measured at 2, 3, 4 and 5 K, at 0.52 GPa. 15
Fig. S9. Field dependence of the magnetisation for **1** measured at 2, 3, 4 and 5 K, at 0.79 GPa. 16
Fig. S10. Field dependence of the magnetisation for **1** measured at 2, 3, 4 and 5 K, at 1.08 GPa. 16
Fig. S11. Plot of the pressure dependence of *D*, as derived from the *ab initio* calculations. 17
Fig. S12. Plot of the pressure dependence of *E*, as derived from the *ab initio* calculations. 17
Fig. S13. Calibration of the results from the *ab initio* calculations to the ambient pressure measurement. 18
Fig. S14. Calibration of the results from the *ab initio* calculations to the ambient pressure measurement. 19

Fig. S1. View of the packing in the unit cell of **1**, showing that none of the four $[Ni(MeDABCO)Cl_3]^+$ cations align. Hydrogen atoms and perchlorate anions have been omitted for clarity.

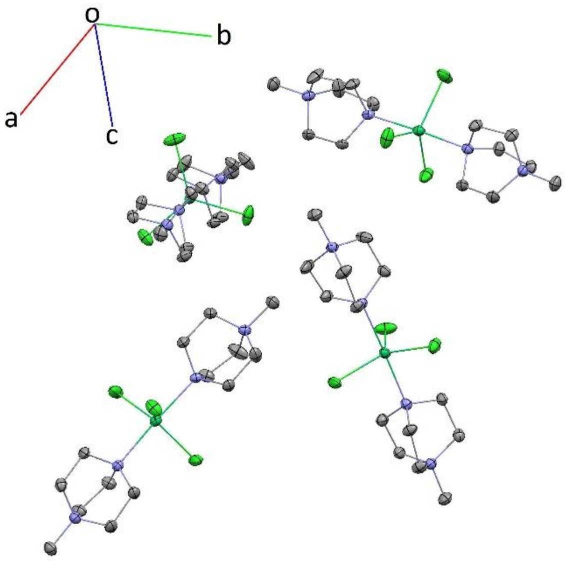

Fig. S2. (Top) Pressure dependence of the unit cell parameters. The solid lines are a guide for the eye. All three axes show a monotonic decrease on increasing pressure until 1.40 GPa. Beyond this point the *a*- and *b*- axes continue to decrease in length with pressure, however the contraction is less pronounced than that observed at lower pressures. The *c*-axis enters a plateau in the pressure range 1.40 – 2.32 GPa, before increasing in length slightly at the highest pressures applied. (Bottom) Pressure dependence of the relative unit cell volume, V/V_0, as a function of pressure. The empty circles represent experimental data. The initial decrease in the unit cell lengths with pressure is reflected in the contraction of the unit cell volume up to an applied pressure of 1.40 GPa. Above this pressure point, the slight increase in the length of the *c*-axis is insufficient to compensate for the contraction along the *a*- and *b*-axes, and the unit cell volume continues to decrease with pressure, although there is a clear change in the compressibility of the compound. The solid red line represents a fit to a second order Birch-Murnaghan equation of state over the range 0 – 1.40 GPa, which yielded a value for the bulk modulus of 11.7(6) GPa. The change in compressibility leads to the significant deviation of the relative unit cell volumes at high pressures from the equation of state. It is difficult to propose a reason for this behaviour at high pressure based on the single crystal structures collected in the lower pressure regime. The crystal packing along the *c*-axis does

not present intermolecular interactions that are distinct from those along the *a*- and *b*-axes. It is worth noting that this change in compressibility arises above the range of pressure achievable in the pressure cell used for the magnetic measurements, and therefore has no bearing on the discussion of the changes in magnetic anisotropy predicted by the theoretical study and subsequent high pressure magnetometry.

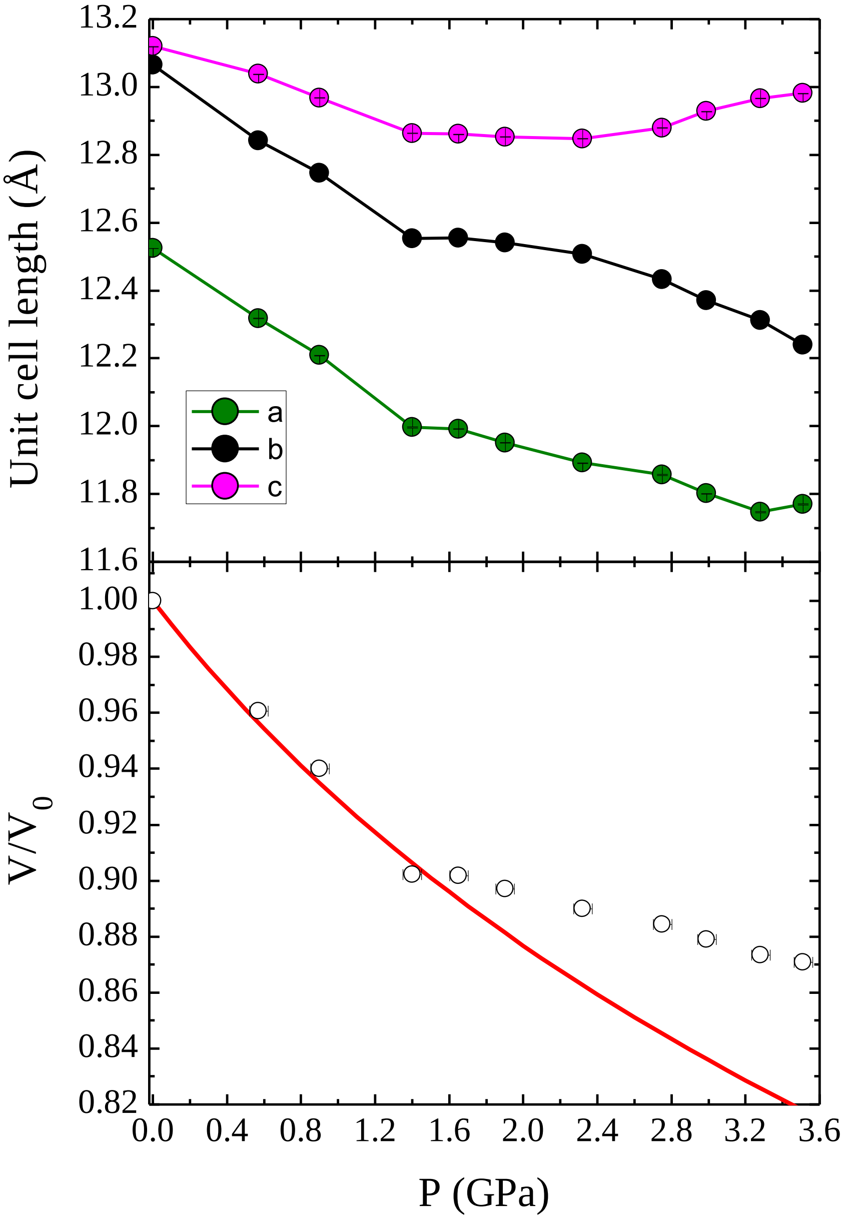

Table S1. Crystallographic data for compound **1**. The ambient pressure data were collected for a single crystal mounted on a Kapton loop, while the high pressure data were collected on a single crystal in a diamond anvil cell under hydrostatic pressure. Above 1.40 GPa there is an apparent change in the compressibility of the compound, and only the unit cell parameters are given.

**Pressure/GPa**	**Ambient**	**0.58**	**0.90**	**1.40**	**1.65**	**1.90**	**2.32**	**2.75**	**2.99**	**3.28**	**3.51**
λ/Å						0.71073					
***T*/K**						293					
**Crystal System**						Orthorhombic					
**Space Group**						$Pca2_1$					
***a*/Å**	12.5175(1)	12.3181(7)	12.2089(9)	11.9968(11)	11.9924(11)	11.9513(10)	11.8918(12)	11.8569(18)	11.8013(14)	11.7470(13)	11.7694(18)
***b*/Å**	13.0820(1)	12.8429(7)	12.7469(8)	12.5527(11)	12.5546(11)	12.5401(9)	12.5071(11)	12.4325(16)	12.3701(13)	12.3120(12)	12.2400(17)
***c*/Å**	13.0989(1)	13.0380(4)	12.9686(5)	12.8642(6)	12.8611(6)	12.8530(6)	12.8479(7)	12.8801(10)	12.9287(8)	12.9666(7)	12.9812(10)
V/Å^3	2145.0(4)	2062.6(2)	2018.2(2)	1937.2(3)	1936.4(3)	1926.3(2)	1910.9(3)	1898.7(4)	1887.4(3)	1875.4(3)	1870.0(4)
Z			4								
D_{calc}/g cm^{-3}	1.607	1.671	1.708	1.780	1.779						
**Reflections**	17781	6211	6037	5214	4999						
**Unique Data**	4863	1738	1740	1583	1596						
R_{int}	0.027	0.029	0.030	0.037	0.035						
R	0.029	0.029	0.031	0.046	0.044						
R_w	0.062	0.071	0.052	0.069	0.070						
S	0.99	1.04	1.00	1.00	0.99						
**Flack Param.**	0.012(14)	0.008(15)	0.014(15)	−0.01(2)	0.02(2)						
ρ_{max}, ρ_{min}/eÅ^{-3}	0.39,−0.36	0.22,−0.19	0.31,−0.31	0.68,−0.92	0.58,−0.80						

Fig. S3. Plot of the bond lengths in the first coordination sphere of the Ni(II) ion as a function of pressure. Colour scheme as given in the key. The solid lines are guides for the eye; for several points the error bars are smaller than the symbols.

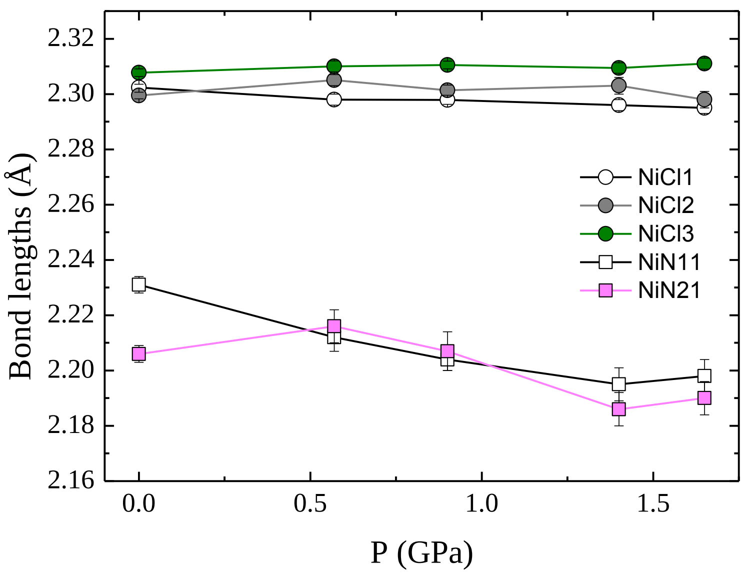

Fig. S4. Plot of the variation in the *trans*- N−Ni−N bond angle as a function of pressure in compound **1**. The solid line is a guide for the eye.

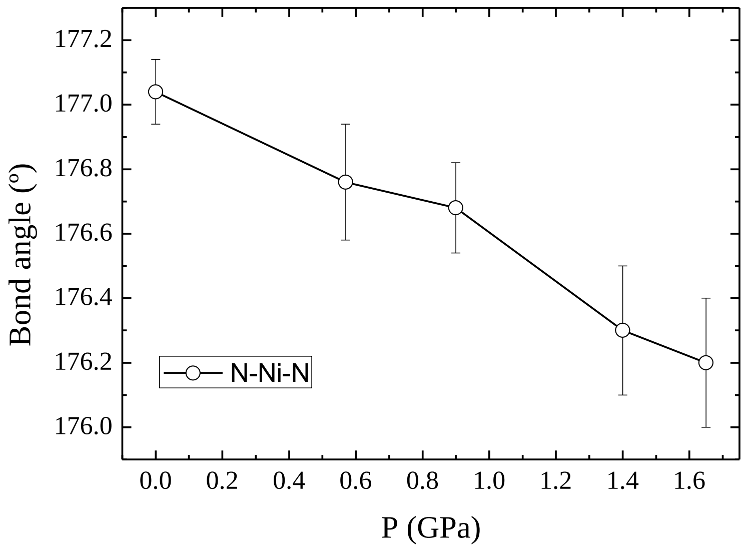

Computational supporting information:

Table S2. Calculated pressure dependence of the parameters *D*, *E*, g_x, g_y, and g_z. Data given are based on NEVPT2 calculations; see Computational details in main paper and following tables.

Complex	*D* (cm^{-1})	*E* (cm^{-1})	g_x	g_y	g_z
1 atm	−399	0.104	1.84	1.84	3.78
0.58 GPa	−347	0.208	1.92	1.92	3.72
0.90 GPa	−317	0.419	1.95	1.97	3.66
1.40 GPa	−264	0.862	2.02	2.04	3.53
1.65 GPa	−264	0.872	2.02	2.04	3.53

Table S3. Comparison of the parameters calculated for the magnetic anisotropy in **1** at ambient pressure using the two different levels of theory.

Ambient pressure		
Calculated parameters	NEVPT2 (EHA)	CASSCF (EHA)
D (cm^{-1})	−399	−409
E/*D*	0.0003	0.0004
g_x	1.84	1.88
g_y	1.84	1.89
g_z	3.78	3.82

Table S4. Details of the major contributing excitations that lead to the *D* and *E* values calculated for compound **1** at ambient pressure. See Fig. 4 in the main text for a schematic representation.

SA-CASSCF states of ambient pressure complex	Major Electronic configurations (%)	Contribution to D (NEVPT2/EHA) (cm^{-1})	Contribution to E (NEVPT2/EHA) (cm^{-1})
Ground state	$d_{yz}^2\ d_{xz}^2\ d_{x2\text{-}y2}^2\ d_{xy}^1\ d_{z2}^1$ (88%)	0.0	0.000
1st excited state	$d_{yz}^2\ d_{xz}^2\ d_{x2\text{-}y2}^1\ d_{xy}^2\ d_{z2}^1$ (88%)	−487.67	0.000
2nd excited state	$d_{yz}^2\ d_{xz}^1\ d_{x2\text{-}y2}^2\ d_{xy}^2\ d_{z2}^1$ (35%)	22.03	19.843
3rd excited state	$d_{yz}^1\ d_{xz}^2\ d_{x2\text{-}y2}^2\ d_{xy}^2\ d_{z2}^1$ (35%)	18.63	−16.970
4th excited state	$d_{yz}^1\ d_{xz}^2\ d_{x2\text{-}y2}^2\ d_{xy}^1\ d_{z2}^2$ (42%)	8.32	−6.134
5th excited state	$d_{yz}^1\ d_{xz}^2\ d_{x2\text{-}y2}^1\ d_{xy}^2\ d_{z2}^2$ (43%)	6.83	4.838

Table S5. Energy of the lowest spin-orbit states for the ambient pressure structure of **1**.

**Spin-orbit/ spin eigenstates**	**Energy (cm^{-1})**	**Ground triplet state (%)**	**1st excited triplet state (%)**
1. $\lvert M_S \rvert = 1$	0.0	64	36
2. $\lvert M_S \rvert = 1$	0.21	64	36
3. $M_S = 0$	398.74	98	2
4. $M_S = 0$	730.15	02	98
5. $\lvert M_S \rvert = 1$	1238.48	36	64
6. $\lvert M_S \rvert = 1$	1249.85	36	64

Table S6. Computed NEVPT2 transition energies and state-wise contribution to the axial and rhombic ZFS parameters at ambient pressure.

Ambient pressure	NEVPT2 energy (cm^{-1})	Contribution to D (cm^{-1})	Contribution to E (cm^{-1})
^{3}F	0.0	0.000	0.000
	335.1	-487.665	0.000
	6435.2	22.031	19.843
	6895.8	18.632	-16.97
	9439.3	8.321	-6.134
	9506.2	6.834	4.838
	11041.4	0.002	-0.002
^{1}D	13211.5	0.008	0.001
	15721.8	0.015	0.000
	16056.2	21.821	0.000
	20950.4	-6.366	-5.499
	21445.5	-6.137	5.306
^{3}P	21835.3	0.071	-0.054
	22524.3	0.086	0.073
	23447.9	-0.001	0.000
^{1}G	26753.7	-0.002	-0.001
	27511.1	0.000	-0.001
	27524.4	-0.032	-0.034
	27811.8	-0.004	-0.004
	27885.2	-0.002	0.002
	31459.9	-2.098	1.188
	31620.0	-1.472	-0.972
	32240.6	-0.350	0.184
	32278.3	0.642	-0.319
^{1}S	57963.0	0.000	0.000

Table S7. Details of the major contributing excitations that lead to the *D* and *E* values calculated for compound **1** at 0.58 GPa.

SA-CASSCF states of 0.58 GPa complex	Major Electronic configurations (%)	Contribution to D (NEVPT2/EHA) (cm^{-1})	Contribution to E (NEVPT2/EHA) (cm^{-1})
Ground state	$d_{yz}^{2}\ d_{xz}^{2}\ d_{x2-y2}^{2}\ d_{xy}^{1}\ d_{z2}^{1}$ (79%)	0.0	0.000
1st excited state	$d_{yz}^{2}\ d_{xz}^{2}\ d_{x2-y2}^{1}\ d_{xy}^{2}\ d_{z2}^{1}$ (79%)	−435.12	0.000
2nd excited state	$d_{yz}^{2}\ d_{xz}^{1}\ d_{x2-y2}^{2}\ d_{xy}^{2}\ d_{z2}^{1}$ (26%)	22.70	−4.500
3rd excited state	$d_{yz}^{1}\ d_{xz}^{2}\ d_{x2-y2}^{2}\ d_{xy}^{2}\ d_{z2}^{1}$ (26%)	19.48	4.819
4th excited state	$d_{yz}^{1}\ d_{xz}^{2}\ d_{x2-y2}^{2}\ d_{xy}^{1}\ d_{z2}^{2}$ (42%)	8.18	−6.188
5th excited state	$d_{yz}^{1}\ d_{xz}^{2}\ d_{x2-y2}^{1}\ d_{xy}^{2}\ d_{z2}^{2}$ (43%)	6.39	4.925

Table S8. Computed NEVPT2 transition energies and state-wise contribution to the axial and rhombic ZFS parameters at 0.58GPa pressure.

0.58 GPa pressure	NEVPT2 energy (cm^{-1})	Contribution to D (cm^{-1})	Contribution to E (cm^{-1})
^3F	0.0	0.000	0.000
	485.9	-435.119	0.000
	6403.5	22.698	-4.499
	6873.6	19.484	4.819
	9218.6	8.177	-6.188
	9307.4	6.391	4.925
	10843.8	0.017	-0.018
^1D	13458	0.021	0.000
	15714.6	0.001	0.000
	16186.3	21.542	0.000
	20888.2	-6.395	1.464
	21371.3	-6.196	-1.507
^3P	21806.3	0.061	0.021
	22436.6	0.072	-0.015
	23208.5	0.000	-0.001
^1G	26786.4	-0.013	-0.011
	27313.1	0.000	-0.002
	27482.7	-0.035	-0.032
	27528.5	-0.002	-0.001
	27670.5	-0.002	-0.001
	31200.5	-2.286	2.06
	31298.8	-2.008	-1.927
	32167.4	-0.184	0.081
	32203.8	1.236	-0.138
^1S	57821.0	0.000	0.000

Table S9. Details of the major contributing excitations that lead to the *D* and *E* values calculated for compound **1** at 0.90 GPa.

SA-CASSCF states of 0.90 GPa complex	Major Electronic configurations (%)	Contribution to D (NEVPT2/EHA) (cm^{-1})	Contribution to E (NEVPT2/EHA) (cm^{-1})
Ground state	$d_{yz}^{2}\ d_{xz}^{2}\ d_{x2-y2}^{2}\ d_{xy}^{1}\ d_{z2}^{1}$ (61%)	0.0	0.000
1st excited state	$d_{yz}^{2}\ d_{xz}^{2}\ d_{x2-y2}^{1}\ d_{xy}^{2}\ d_{z2}^{1}$ (61%)	-403.48	0.000
2nd excited state	$d_{yz}^{2}\ d_{xz}^{1}\ d_{x2-y2}^{2}\ d_{xy}^{2}\ d_{z2}^{1}$ (22%)	22.26	-14.861
3rd excited state	$d_{yz}^{1}\ d_{xz}^{2}\ d_{x2-y2}^{2}\ d_{xy}^{2}\ d_{z2}^{1}$ (27%)	19.60	14.040
4th excited state	$d_{yz}^{2}\ d_{xz}^{1}\ d_{x2-y2}^{2}\ d_{xy}^{1}\ d_{z2}^{2}$ (47%)	7.93	-6.319
5th excited state	$d_{yz}^{1}\ d_{xz}^{2}\ d_{x2-y2}^{2}\ d_{xy}^{1}\ d_{z2}^{2}$ (46%)	5.87	4.736

Table S10. Computed NEVPT2 transition energies and state-wise contribution to the axial and rhombic ZFS parameters at 0.90GPa pressure.

0.90 GPa pressure	NEVPT2 energy (cm^{-1})	Contribution to D (cm^{-1})	Contribution to E (cm^{-1})
^{3}F	0.0	0.000	0.000
	589	-403.476	0.000
	6504.8	22.264	-14.861
	7023.5	19.602	14.040
	9471.2	7.926	-6.319
	9575.5	5.871	4.736
	11093.3	0.021	-0.019
^{1}D	13381.8	0.076	0.000
	15714.5	0.001	0.000
	16287.3	21.404	0.000
	21005.2	-6.353	4.505
	21560.2	-6.208	-4.500
^{3}P	21895.8	0.068	0.056
	22634.8	0.073	-0.050
	23465.8	0.000	-0.001
^{1}G	26841.1	-0.031	-0.015
	27448.3	0.007	-0.002
	27613.2	-0.024	-0.022
	27815.0	-0.002	0.001
	27971.3	-0.002	-0.002
	31517.3	-2.051	1.918
	31583.6	-1.771	-1.825
	32319.0	-0.371	0.231
	32392.8	1.023	-0.249
^{1}S	58001.2	0.000	0.000

Table S11. Details of the major contributing excitations that lead to the *D* and *E* values calculated for compound **1** at 1.40 GPa.

SA-CASSCF states of 1.40 GPa complex	Major Electronic configurations (%)	Contribution to D (NEVPT2/EHA) (cm^{-1})	Contribution to E (NEVPT2/EHA) (cm^{-1})
Ground state	$d_{yz}^2\ d_{xz}^2\ d_{x2-y2}^2\ d_{xy}^1\ d_{z2}^1$ (51%)	0.0	0.000
1st excited state	$d_{yz}^2\ d_{xz}^2\ d_{x2-y2}^1\ d_{xy}^2\ d_{z2}^1$ (51%)	-345.54	0.000
2nd excited state	$d_{yz}^2\ d_{xz}^1\ d_{x2-y2}^2\ d_{xy}^2\ d_{z2}^1$ (32%)	21.81	-19.364
3rd excited state	$d_{yz}^1\ d_{xz}^2\ d_{x2-y2}^2\ d_{xy}^2\ d_{z2}^1$ (32%)	19.60	17.933
4th excited state	$d_{yz}^2\ d_{xz}^1\ d_{x2-y2}^2\ d_{xy}^1\ d_{z2}^2$ (48%)	7.45	-6.558
5th excited state	$d_{yz}^1\ d_{xz}^2\ d_{x2-y2}^2\ d_{xy}^1\ d_{z2}^2$ (49%)	5.03	4.459

Table S12. Computed NEVPT2 transition energies and state-wise contribution to the axial and rhombic ZFS parameters at 1.40 GPa pressure.

1.40 GPa pressure	NEVPT2 energy (cm^{-1})	Contribution to D (cm^{-1})	Contribution to E (cm^{-1})
^3F	0.0<br>812.5<br>6694.3<br>7298.8<br>9889.3<br>10033.3<br>11521.3	0.000<br>-345.535<br>21.813<br>19.602<br>7.451<br>5.033<br>0.025	0.000<br>0.000<br>-19.364<br>17.933<br>-6.558<br>4.459<br>-0.022
^1D	13292.9<br>15715.2<br>16509.1<br>21210.5<br>21895.1	0.171<br>0.002<br>21.116<br>-6.299<br>-6.22	0.000<br>0.000<br>0.000<br>5.753<br>-5.744
^3P	22067.2<br>23009.1<br>23892.5	0.078<br>0.073<br>0.001	0.076<br>-0.067<br>-0.001
^1G	26966.2<br>27654.0<br>27869.9<br>28266.1<br>28496.6<br>32043.8<br>32114.4<br>32558.3<br>32764.1	-0.036<br>0.020<br>-0.047<br>-0.002<br>-0.001<br>-1.510<br>-1.492<br>-0.854<br>0.825	-0.016<br>-0.001<br>0.020<br>0.000<br>-0.003<br>1.383<br>-1.427<br>0.407<br>-0.302
^1S	58327.9	0.001	0.000

Table S13. Details of the major contributing excitations that lead to the *D* and *E* values calculated for compound **1** at 1.65 GPa.

SA-CASSCF states of 1.65 GPa complex	Major Electronic configurations (%)	Contribution to D (NEVPT2/EHA) (cm^{-1})	Contribution to E (NEVPT2/EHA) (cm^{-1})
Ground state	$d_{yz}^2\ d_{xz}^2\ d_{x2-y2}^2\ d_{xy}^1\ d_{z2}^1$ (50%)	0.0	0.000
1st excited state	$d_{yz}^2\ d_{xz}^2\ d_{x2-y2}^1\ d_{xy}^2\ d_{z2}^1$ (50%)	-346.40	0.000
2nd excited state	$d_{yz}^2\ d_{xz}^1\ d_{x2-y2}^2\ d_{xy}^2\ d_{z2}^1$ (32%)	21.82	-19.577
3rd excited state	$d_{yz}^1\ d_{xz}^2\ d_{x2-y2}^2\ d_{xy}^2\ d_{z2}^1$ (32%)	19.72	19.428
4th excited state	$d_{yz}^2\ d_{xz}^1\ d_{x2-y2}^2\ d_{xy}^1\ d_{z2}^2$ (48%)	7.50	-6.750
5th excited state	$d_{yz}^1\ d_{xz}^2\ d_{x2-y2}^2\ d_{xy}^1\ d_{z2}^2$ (49%)	5.01	4.533

Table S14. Computed NEVPT2 transition energies and state-wise contribution to the axial and rhombic ZFS parameters at 1.65 GPa pressure.

1.65 GPa pressure	NEVPT2 energy (cm^{-1})	Contribution to D (cm^{-1})	Contribution to E (cm^{-1})
^3F	0.0<br>808.4<br>6679.4<br>7285.8<br>9840.2<br>9986.3<br>11478.3	0.000<br>-346.396<br>21.818<br>19.721<br>7.501<br>5.006<br>0.031	0.000<br>0.000<br>-19.577<br>18.201<br>-6.750<br>4.533<br>-0.028
^1D	13319.5<br>15712.3<br>16502.4<br>21187.2<br>21876.2	0.183<br>0.001<br>21.10<br>-6.297<br>-6.226	0.000<br>0.000<br>0.000<br>5.790<br>-5.783
^3P	22036.4<br>22985.1<br>23849.4	0.076<br>0.071<br>0.002	0.075<br>-0.065<br>-0.002
^1G	26942.7<br>27615.9<br>27856.8<br>28203.6<br>28435.7<br>31990.6<br>32048.4<br>32551.4<br>32734.3	-0.042<br>0.020<br>-0.048<br>-0.003<br>-0.003<br>-1.507<br>-1.431<br>-0.85<br>0.758	-0.023<br>-0.002<br>0.021<br>0.000<br>-0.004<br>1.381<br>-1.432<br>0.454<br>-0.336
^1S	58289.5	0.001	0.000

Fig. S5. The temperature dependence of the molar magnetic susceptibility, $\chi_M T$, for **1**, measured at 0.52 GPa. The solid red line represents a simulation of the data using the parameters given in the main text.

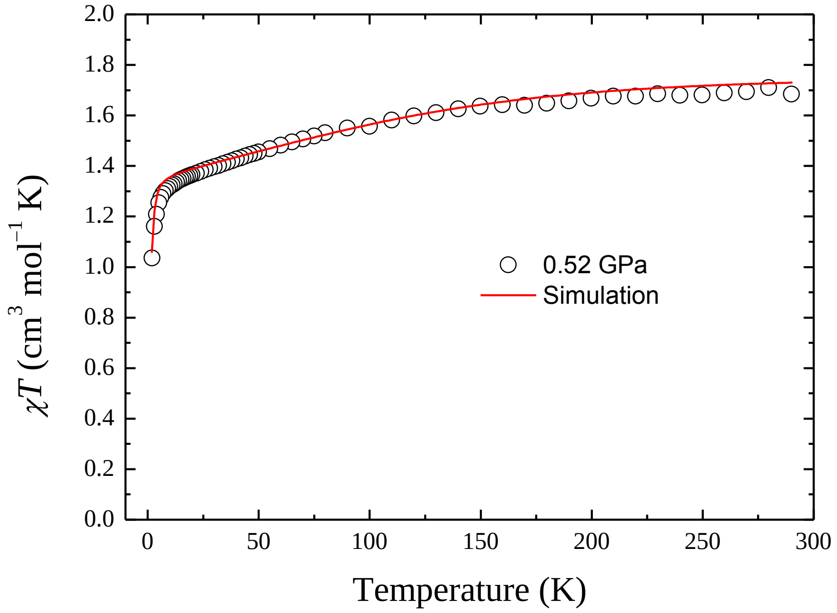

Fig. S6. The temperature dependence of the molar magnetic susceptibility, $\chi_M T$, for **1**, measured at 0.79 GPa. The solid red line represents a simulation of the data using the parameters given in the main text.

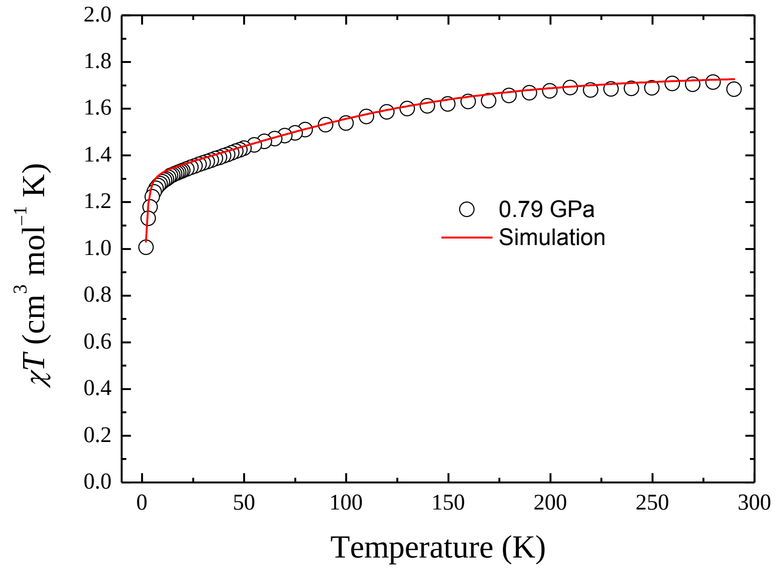

Fig. S7. Field dependence of the magnetisation for **1** measured at 2, 3, 4 and 5 K, at ambient pressure. The solid red line represents a simulation of the data using the parameters given in the main text.

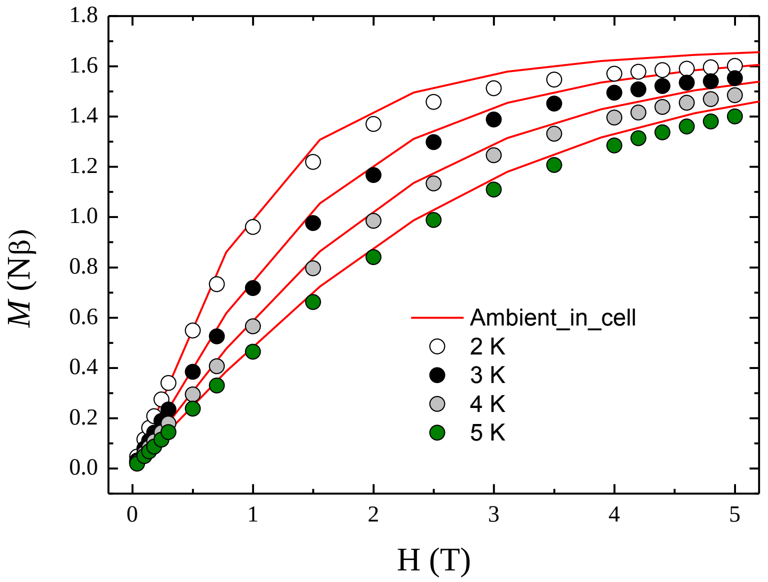

Fig. S8. Field dependence of the magnetisation for **1** measured at 2, 3, 4 and 5 K, at 0.52 GPa. The solid red line represents a simulation of the data using the parameters given in the main text.

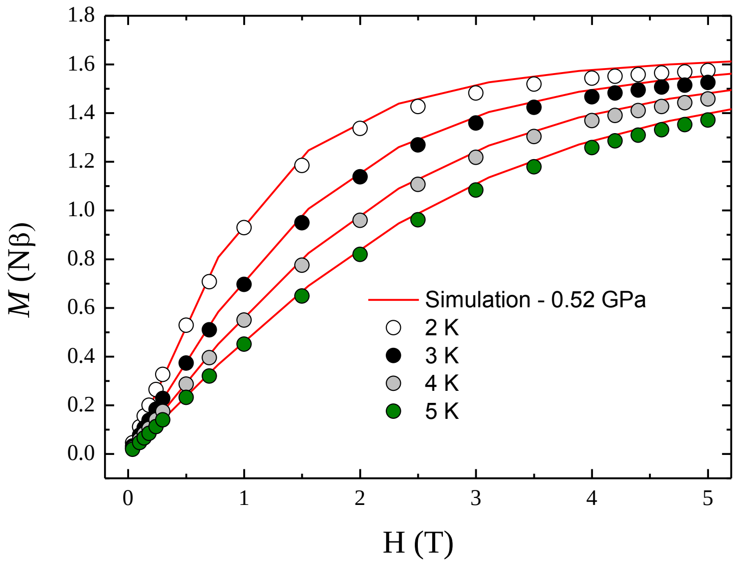

Fig. S9. Field dependence of the magnetisation for **1** measured at 2, 3, 4 and 5 K, at 0.79 GPa. The solid red line represents a simulation of the data using the parameters given in the main text.

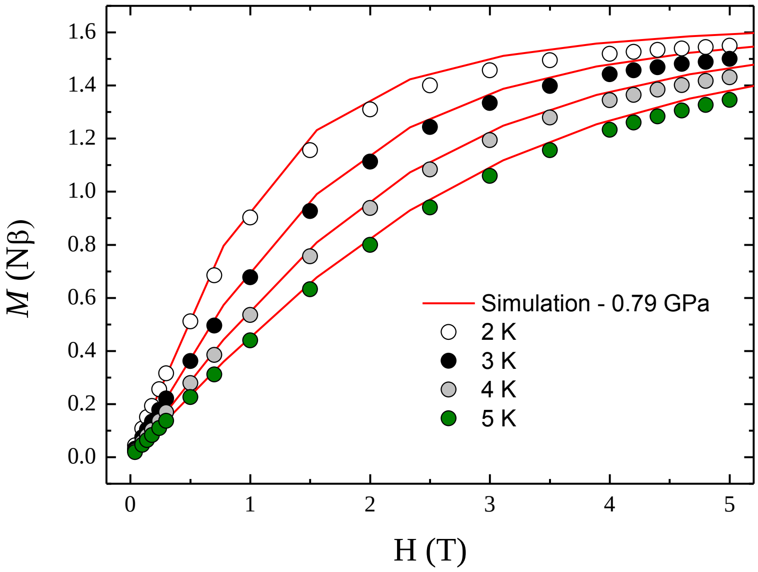

Fig. S10. Field dependence of the magnetisation for **1** measured at 2, 3, 4 and 5 K, at 1.08 GPa. The solid red line represents a simulation of the data using the parameters given in the main text.

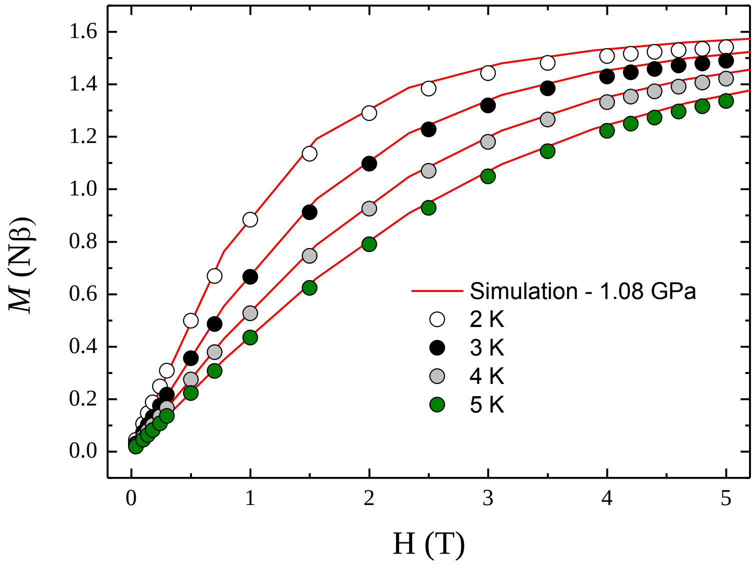

Fig. S11. Plot of the pressure dependence of *D*, as derived from the *ab initio* calculations. The red line is straight line fit of these data, which was used to extract the values of *D* used in the simulations of the magnetic data (see main text).

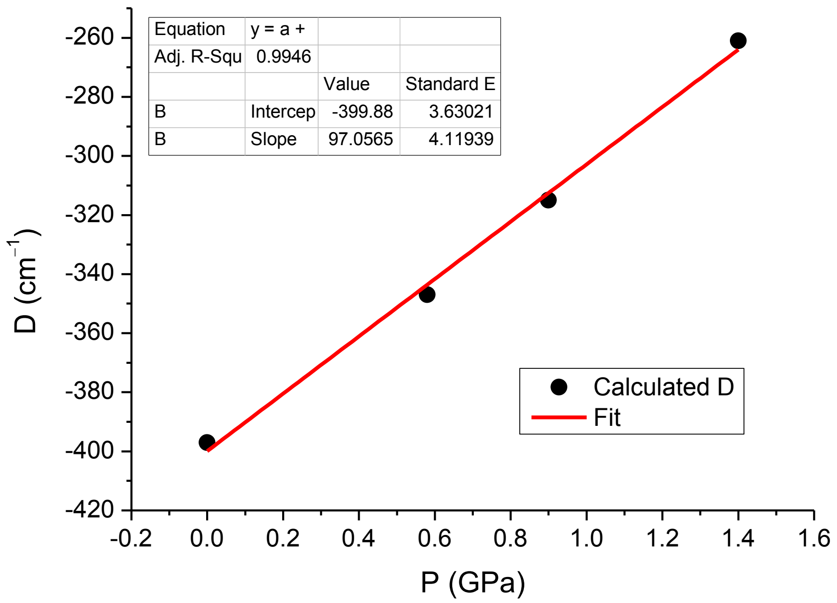

Fig. S12. Plot of the pressure dependence of *E*, as derived from the *ab initio* calculations. The red line is the straight line fit of these data, however, this fit appears unsuitable. Therefore, an exponential fit was applied and used to extract the values of *E* used in the simulations of the magnetic data (see main text).

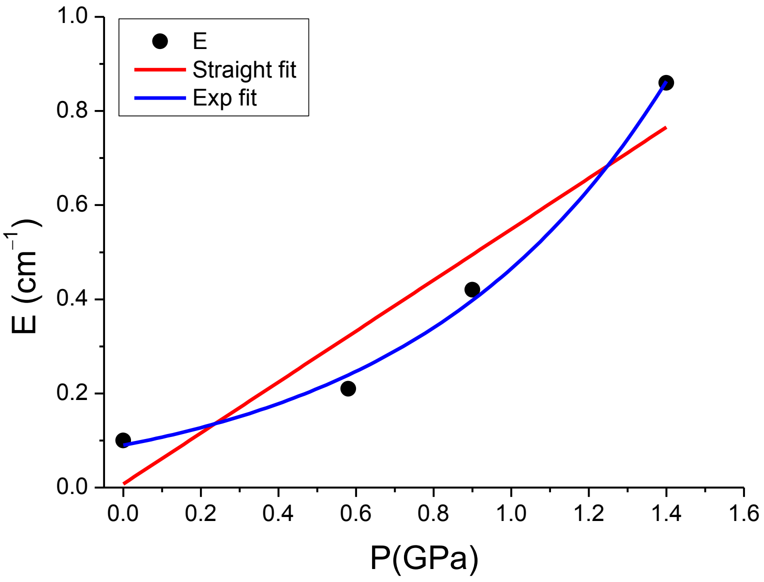

Fig. S13. Calibration of the results from the *ab initio* calculations to the ambient pressure measurement. Black circles show the results of the *ab initio* calculations for g_z. The calculated g_z at ambient pressure was normalised to the value determined by HFEPR, and the other data points normalised accordingly. The red line is the straight line fit of these data, which was used to extract the values of g_z used in the simulations of the magnetic data (see main text).

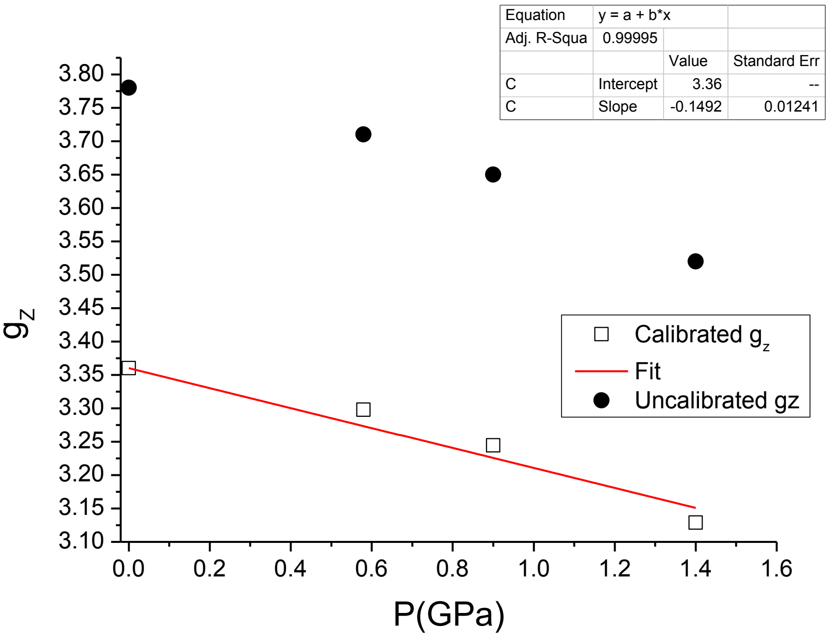

Fig. S14. Calibration of the results from the *ab initio* calculations to the ambient pressure measurement. Black and white circles show the results of the *ab initio* calculations for g_y and g_x. The calculated g_x and g_y at ambient pressure were normalised to the value determined by a fit of the ambient pressure magnetic data, and the other data points normalised accordingly. The red lines are the straight line fits of these data, which were used to extract the values of g_x and g_y used in the simulations of the magnetic data (see main text).

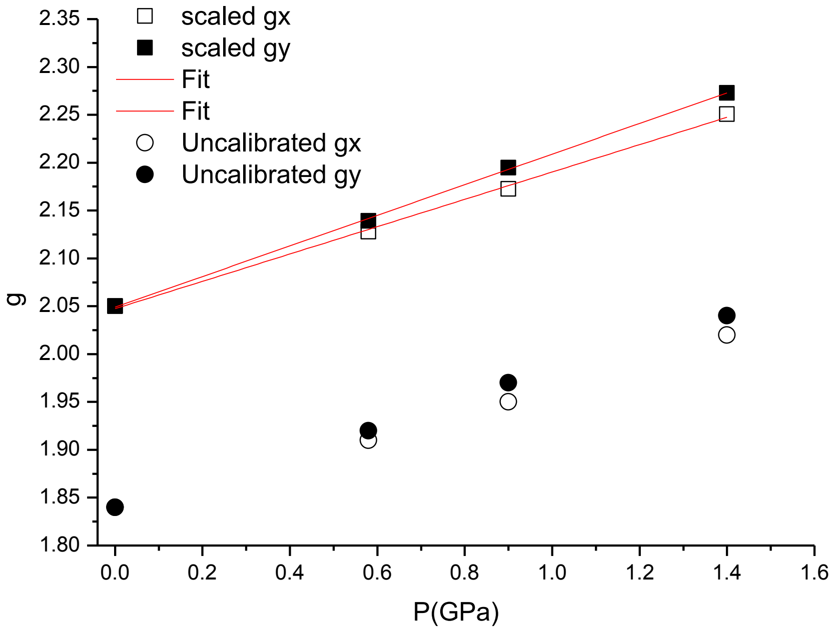

Supplement: Supplementary file 1 [file SC-009-C7SC04460G-s001.pdf]
